# What Controls the Quality of Photodynamical Simulations? Electronic Structure Versus Nonadiabatic Algorithm

**DOI:** 10.1021/acs.jctc.3c00908

**Published:** 2023-11-08

**Authors:** Jiří Janoš, Petr Slavíček

**Affiliations:** Department of Physical Chemistry, University of Chemistry and Technology, Technická 5, 16628 Prague 6, Czech Republic

## Abstract

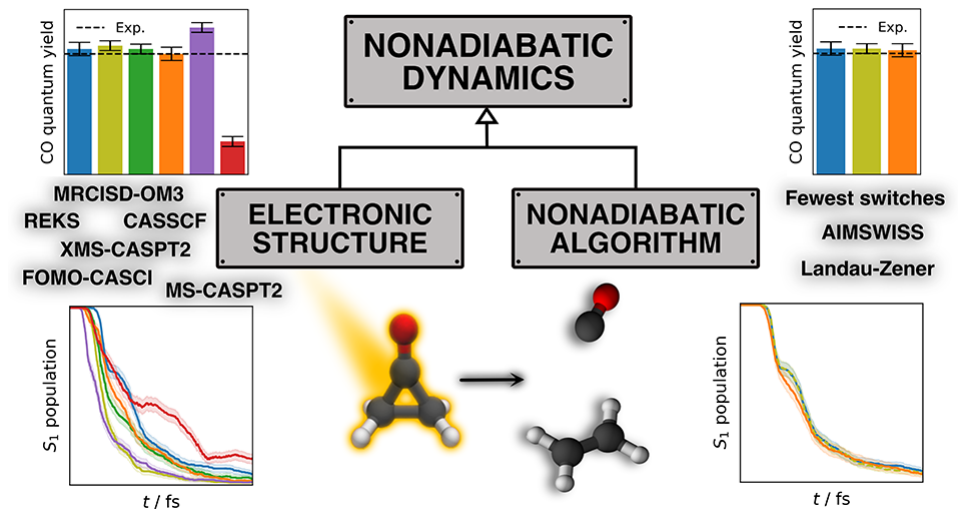

The field of nonadiabatic dynamics has matured over the last decade with a range of algorithms and electronic structure methods available at the moment. While the community currently focuses more on developing and benchmarking new nonadiabatic dynamics algorithms, the underlying electronic structure controls the outcome of nonadiabatic simulations. Yet, the electronic-structure sensitivity analysis is typically neglected. In this work, we present a sensitivity analysis of the nonadiabatic dynamics of cyclopropanone to electronic structure methods and nonadiabatic dynamics algorithms. In particular, we compare wave function-based CASSCF, FOMO-CASCI, MS- and XMS-CASPT2, density-functional REKS, and semiempirical MRCI-OM3 electronic structure methods with the Landau–Zener surface hopping, fewest switches surface hopping, and *ab initio* multiple spawning with informed stochastic selection algorithms. The results clearly demonstrate that the electronic structure choice significantly influences the accuracy of nonadiabatic dynamics for cyclopropanone even when the potential energy surfaces exhibit qualitative and quantitative similarities. Thus, selecting the electronic structure solely on the basis of the mapping of potential energy surfaces can be misleading. Conversely, we observe no discernible differences in the performance of the nonadiabatic dynamics algorithms across the various methods. Based on the above results, we discuss the present-day practice in computational photodynamics.

## Introduction

Techniques of nonadiabatic dynamics have become an almost routine tool in theoretical investigations of the ultrafast photodynamics of small- to medium-sized chromophores. The research area was greatly accelerated by the continuous development of ultrafast time-resolved laser spectroscopies, with the time resolution of femtoseconds or even hundreds of attoseconds now available.^1−5^ The experiments bring new observables, ranging from the UV and IR transient absorption, Raman spectroscopy, or ultrafast electron diffraction to a fascinating area of time-resolved photoemission spectroscopies, offering direct snapshots of the electronic structure of chromophores both in the gas and liquid phase.^6−8^

As the direct (on-the-fly) nonadiabatic dynamics have gradually become a user technology, with different packages being made available,^9−11^ a wide range of possibilities is now open for researchers. However, this also brings the burden of the right choice and control of the outcome. Any simulation requires selecting two main components: the algorithm for coupled electron–nuclear motion (nonadiabatic dynamics algorithm) and the electronic structure method used for the underlying potential energy surfaces and couplings. To keep simulations feasible, a higher quality electronic structure often aligns with a simpler nonadiabatic algorithm and vice versa. The choice is paralleled to the usual dilemma in quantum chemistry, balancing the correlation energy and basis set size. Yet unlike in quantum chemistry, the “focal point” is typically never reached in photodynamics simulations. The decision in photodynamics is led by experience, previous expertise, code availability, and time feasibility.

Multiple schemes for coupled electron–nuclear dynamics have been proposed over the last decades.^12^ The most common are trajectory-based approaches which replace the nuclear wave function with a swarm of classically evolved trajectories.^13^ Among them, the trajectory surface hopping (TSH) methods are the most frequently used.^14^ As a consequence of the classical treatment, quantum effects, such as tunneling or interference phenomena, are neglected. The missing electronic transitions are added ad hoc via several flavors of hopping schemes. The fewest-switches hopping algorithm proposed by Tully^15^ has become a workhorse of nonadiabatic dynamics for its simplicity and efficiency, although it suffers from some well-known problems such as the lack of decoherence.^16,17^ Various improvements and patches for decoherence were suggested, e.g., augmented fewest switches surface hopping,^18−20^ exact factorization decoherence scheme,^17,21^ Ehrenfest-based algorithms,^22−24^ or an empirical treatment.^16^ An appealing and simpler version of the surface hopping algorithm is based on the Landau–Zener formula modified for the adiabatic states.^25−27^ This technique does not require nonadiabatic couplings to evaluate the transition probabilities and its performance seems to be satisfactory under certain conditions, with superior stability over other methods.^28,29^ Another trajectory-based approach exploits the Ehrenfest theorem which evolves the trajectories on average potential energy surfaces.^24,30^ Although this approach suffers from well-known deficiencies for systems with multiple reaction channels, it might still be a method of choice for dynamics in a high-manifold of electronic states or, e.g., in real-time quantum chemistry.^31−33^

Going beyond the trajectory-based approaches, a successful strategy is based on expanding the nuclear wave function into a localized basis set, typically Gaussian wave packets. The basis set localization is dictated by the local character of the quantum chemistry codes. In the simplest and most efficient methods from this family, the *ab initio* multiple spawning (AIMS) schemes, one drives the Gaussian wave packets by classical dynamics.^34−36^ AIMS offers the flexibility of adaptive basis sets, typically “spawned” in the nonadiabatic region. Unlike TSH, AIMS inherently accounts for decoherence effects and nonadiabatic transitions. Yet the advantage comes at a price; AIMS is considerably more demanding and prone to instabilities. To overcome these drawbacks, AIMS with informed stochastic selection (AIMSWISS) was developed.^37^ AIMSWISS combines AIMS with the stochastic selection idea inherent to TSH, resulting in a method that lies between the two. The advantage of the AIMSWISS method is often AIMS-quality results for a cost close to TSH.^38^ More advanced techniques for coupled electron–nuclear dynamics then exploit the time-dependent variational principle to drive the nuclear wave functions, e.g., the dd-vMCG method.^39,40^ These approaches are already highly demanding and require a gradual buildup of a semianalytical potential energy surface.^41^ Thus, their applicability to medium-sized molecules is limited.

The menu for electronic structure methods seems at first almost infinite yet the method availability is limited for nonadiabatic dynamics in excited states.^13,42−44^ We need methods that would be globally reliable, including also the treatment of dissociative states. Furthermore, nonadiabatic couplings are required for most of the algorithms. Popular and thrifty single-reference methods such as time-dependent density functional theory or adiabatic diagrammatic construction techniques are sometimes used for specific applications,^45,46^ with the possibility to design tailored approaches such as spin-flip density functional theory (DFT),^47,48^ ΔSCF,^49,50^ hole–hole Tamm-Dancoff DFT,^51^ or constrained DFT.^52^ However, multireference methods are generally needed. They typically start with the complete active space self-consistent field method (CASSCF), adding dynamical correlation either via second-order perturbation theory (CASPT2) or multireference configuration interaction (MRCI).^44^ Efficient *ab initio* alternatives of CASSCF, such as the floating occupation molecular orbital-complete active space configuration interaction (FOMO-CASCI) method, have also found applications.^53−55^ Semiempirical versions of these multireference approaches, such as MRCI with the orthogonalization-corrected Hamiltonians (OMx)^56^ or FOMO-CI,^57^ offer cost-efficient alternatives. Additionally, DFT can be modified to describe multireference systems. Apart from the above-mentioned spin-flip approaches,^47^ the restricted ensemble-referenced Kohn–Sham (REKS) method has been suggested as an efficient approach. It has already been applied to the photodissociation of cyclopropanone.^58^ Recently, also hybrid methods combining *ab initio* and DFT approaches have been developed; a prominent example is the multiconfigurational pair-density functional theory (MC-PDFT) with its multistate variants.^59−61^

In the computational photodynamics community, most of the attention is focused on developing and defending new technologies for coupled electron–nuclear dynamics. This is understandable, as the electronic structure theory is considered to be an external input. As a result, the researchers in the field are well aware of the possible sources of errors caused by different nonadiabatic algorithms, unlike in the case of electronic structure. The impact of different electronic structure methods is difficult to infer from inspecting important points on potential energy surfaces. Since running dynamics with several electronic structure methods is costly, most of the photodynamics studies rely on a single electronic structure method without controlling the systematic error. Thus, it remains unclear which of these two nonadiabatic dynamics components, coupled electron–nuclear algorithm or electronic structure method, is the accuracy-determining one.

Here, we attempt to assess the errors caused by standard nonadiabatic dynamics algorithms and electronic structure methods and emphasize that we should primarily focus on the electronic structure part of the problem. We systematically evaluate the performance of different algorithms of coupled electron–nuclear dynamics (FSSH, LZSH, and AIMSWISS) and electronic structure methods (CASSCF, CASPT2, FOMO-CASCI, OM3, and REKS) to compare their impact on the resulting population traces and quantum yields. We also analyze the transition regions using an advanced statistical technique called multidimensional scaling. As a test molecule, cyclopropanone, which dissociates into carbon monoxide and ethene upon irradiation with UV light, is used.

## Methods

### Electronic Structure Methods

Throughout the work, we applied a series of multiconfigurational methods, namely, state-averaged CASSCF (SA-CASSCF), state-specific CASPT2 (SS-CASPT2), multistate CASPT2 (MS-CASPT2), extended multistate CASPT2 (XMS-CASPT2), multireference configuration interaction (MRCI), floating occupation molecular orbitals based complete active space configuration interaction (FOMO-CASCI), multireference configuration interaction with singles and doubles/orthogonalization-corrected method 3 (MR-CISD/OM3), and state-interaction state-average REKS (SI-SA-REKS). For mapping the potential energy surfaces and nonadiabatic dynamics, we used an active space comprising 8 electrons in 7 orbitals—two σ_CC_, π_CO_, n_O_, π_CO_^*^, and two σ_CC_^*^—denoted as (8,7) for all the above methods except for SI-SA-REKS. Here, the only currently implemented active space is (2,2) which contained only n_O_ and π_CO_^*^ orbitals. The (8,7) active space was selected based on previous studies,^62^ automated selection in the AutoCAS code,^63^ and benchmarking of potential energy surfaces and absorption spectra. The orbitals composing both (8,7) and (2,2) active spaces are depicted in Supporting Information. For the ground state sampling, we applied the (2,4) active space as a cost-effective alternative for the active space of (8,7). The 6-31g* basis set was used for most of the calculations. The choice corresponds to a basis set used in previous studies.^28,58,64^ The only exception is the MR-CISD/OM3 method, where a pseudominimal basis set is used by default, and the CASPT2 simulations performed in BAGEL package, where this basis set is not available and was replaced by a slightly more extended cc-pVDZ basis. All the CASPT2 calculations were performed with a level shift of 0.5 a.u. The influence of the shift value was tested to be negligible over a wide range of reasonable values. All calculations considered only the S_0_ and S_1_ states, equally weighted in the state-averaging procedure of SA-CASSCF.

The SA-CASSCF, CASPT2 (for mapping the potential energy surfaces), and MRCI calculations were performed in the Molpro 2012.1 code.^65,66^ The CASPT2 calculations for nonadiabatic simulations were performed in the BAGEL package.^67^ Semiempirical MR-CISD/OM3 calculations were done in the MNDO v7.0 code.^68^ The SI-SA-REKS and FOMO-CASCI energies were calculated in the TeraChem v1.9 package.^69,70^

### Coupled Electron–Nuclear Dynamics

The initial positions and momenta for the nonadiabatic simulations were sampled via ground-state molecular dynamics with constant excitation energy.^71^ This approach mimics excitation by a continuous-wave laser within the nuclear ensemble approach as it samples only geometries with constant excitation energy, i.e., in resonance with the laser field.^72^ We selected six different excitation energies covering the whole spectrum and sampled 100 geometries and momenta for each interval; thus, 600 points in the phase space were used in total. The advantage of this approach compared to standard harmonic Wigner sampling is that we can efficiently cover also tails of the spectra by tuning the excitation energy. The potential energies were obtained using the SA-CASSCF(2,4) method. The time step was set to 20 a.u and the thermal effects were included via the Nosé–Hoover thermostat^73^ with the temperature set to 300 K. For more details about the sampling of the initial conditions, see Supporting Information.

For the subsequent nonadiabatic dynamics, we used three methods: Landau–Zener surface hopping (LZSH), fewest switches surface hopping (FSSH), and AIMSWISS. The algorithms are outlined in the Introduction and the references therein. For the surface hopping techniques, the time step of 5 a.u. was used. The energy decoherence correction for FSSH was applied with a value of 0.1 a.u.^16^ For the AIMSWISS simulations, the time step of 20 a.u. was adapted to 5 a.u. and further in the coupling regions to secure the correct propagation and transfer of the amplitude in the coupling regions. The coupling threshold to enter the spawning mode was set to 3 a.u.

All the ground-state and surface-hopping dynamics were done in our in-house code ABIN.^74^ The AIMSWISS simulations were done with a modified version of the FMS90/Molpro interface.^75^

### Analysis of Simulations

The time evolution of the electronic populations was calculated as an average over an ensemble of trajectories (weighted average over trajectory basis functions in the AIMSWISS framework) alive at the moment. In other words, a given trajectory contributes to the population only up to the time that it fails. If the trajectory failed in the ground state, it was considered to stay there until the end of the simulation time, as no hopping back to the excited state was ever observed. We note that the treatment of the prematurely ending trajectories is not well established in the field. However, since we benchmark methods, it is consistency that is important. The estimate of error bars is based on the binomial distribution as , where *N*(*t*) is the number of active trajectories at time *t*, *p*(*t*) is the S_1_ population at time *t*, and *z* = 1.96 corresponds to a 95% confidence interval.

In order to obtain lifetimes, the populations were fitted to a delayed exponential decay function
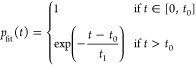
1where *t*_0_ and *t*_1_ are the fitted parameters. The *t*_0_ time represents the delay between the excitation and the beginning of the exponential decay with the lifetime *t*_1_. The total lifetime is evaluated as τ = *t*_0_ + *t*_1_ and corresponds to the time when the fitted excited-state population decreases to 1/e.

The quantum yields of CO photodissociation were evaluated as  where *N*_0_ is the number of simulations and *N*_0_^dis^ is the number of simulations where CO was released. Only the simulations ending in the ground state were considered. The statistical error of ϕ_p_ was estimated from the binomial distribution as  where *z* = 1.96 to express the 95% confidence interval.

We further analyzed transition regions, i.e., hopping/spawning geometries. These geometries provide information about the intersection seams visited in the dynamics. To analyze them, we applied a multidimensional scaling (MDS) statistical approach. MDS takes high-dimensional data (geometries of molecules in our case) and represents them in a low-dimensional space, similar to principal component analysis. The projection to a low-dimensional space is nonlinear and preserves distances between data points as much as possible, which makes MDS advantageous when sets of data are compared.

The MDS procedure was implemented as follows: first, we aligned the molecular geometries so that the root-mean-square deviation was minimized. Then, Cartesian distances between the aligned geometries were calculated. Only carbon and oxygen atoms were used for alignment and distance calculations, because hydrogen atoms only increased the noise while bringing no relevant information. Next, the *m* × *m* pairwise dissimilarity matrix **D** was constructed with *m* being the number of geometries. The elements of matrix **D** are squared distances between the data points. Then, a matrix **B** was constructed from **D** as
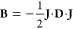
2where **J** is defined with the identity matrix **I** (diagonal elements are equal to one) and the matrix of ones **1** (all elements are equal to one) as
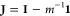
3

Finally, the matrix **B** was diagonalized to obtain a set of eigenvectors *e⃗*_i_ and corresponding eigenvalues ξ_*i*_. This set was reordered from the largest to the smallest eigenvalue, as the eigenvalues are proportional to the variance covered by their corresponding coordinates. The representation of the *i*-the original data point in the new reduced coordinates (RCs) *f⃗*_i_ is then constructed as
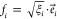
4

More details on MDS applied to molecular geometries can be found in the work of Li et al.^76^ where it was used to analyze the photoisomerization dynamics of phytochromobilin chromophore.

## Results

We begin with the overall presentation of the cyclopropanone photodecomposition mechanism, reporting a new rare pathway theoretically discovered in this work. Next, we map the potential energy surface along relevant coordinates for all electronic structure methods applied in the comparison and highlight the distinct features among the methods. We emphasize the spurious behavior of the perturbative approaches near conical intersections. A major part of the work focuses on comparing the electronic structure methods and nonadiabatic dynamics algorithms for several quantities, being either observables or other properties. The comparison is based on excited-state populations, quantum yields, and the transition region analysis. The effect of the small active space for the SI-SA-REKS method is also inspected. Finally, we elaborate on the impact of a conical intersection description in perturbative approaches on nonadiabatic dynamics.

### Mechanism of Photochemical Decomposition

**Figure 1:**
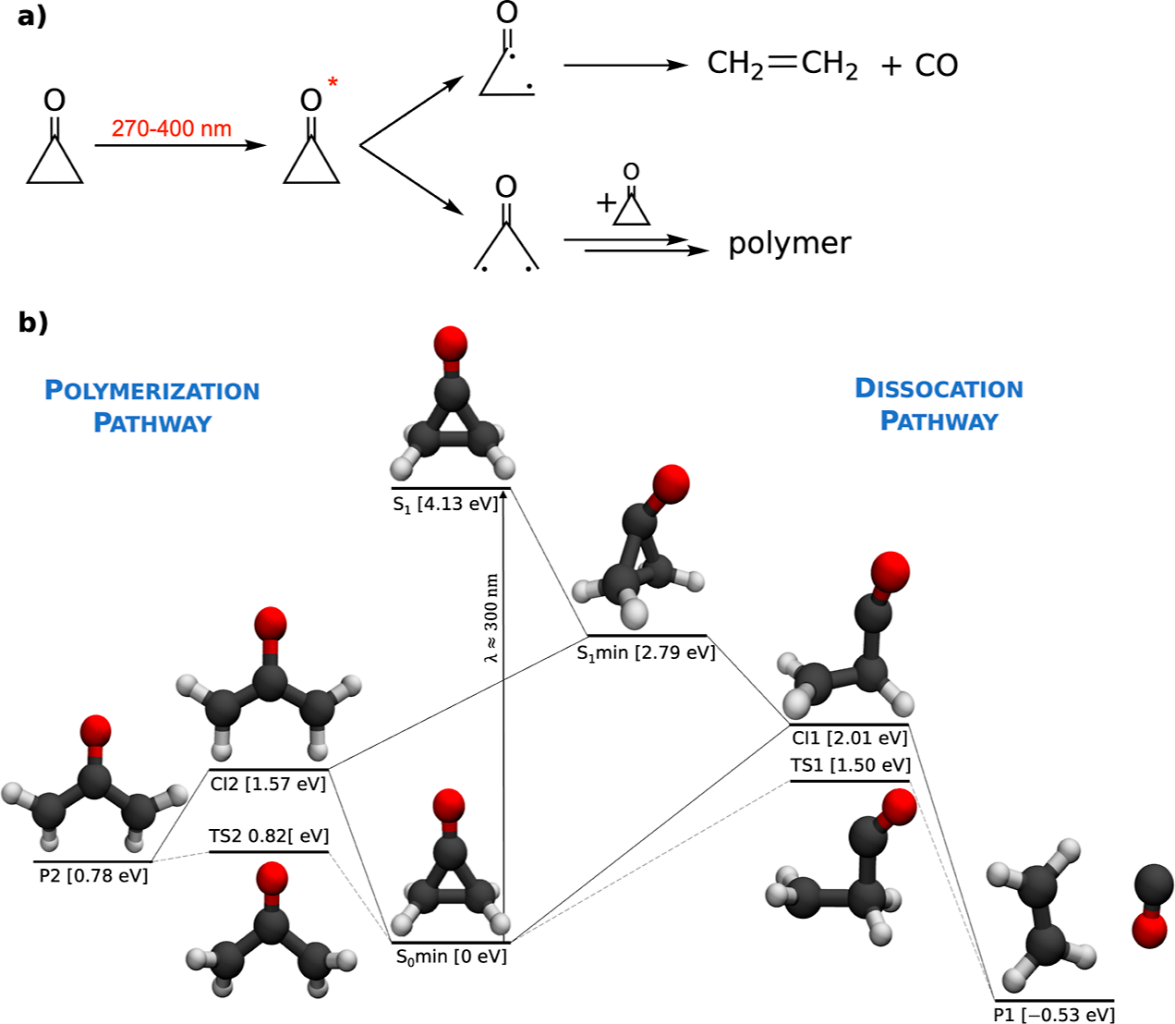
(a) Scheme of the two reaction pathways of cyclopropanone photodecomposition. (b) Mapping of the important points on the potential energy surface calculated at the SA-CASSCF(8,7) level. Solid lines correspond to photochemical pathways, while dashed lines represent possible thermal reactions in the ground state. Recalculation of the energies with MRCI and XMS-CASPT2 is available in Supporting Information.

The photochemistry of cyclopropanone is driven by its strained three-membered ring, leading to ultrafast dynamics in the excited states. The process is initiated by an excitation into the S_1_ state with an absorption maximum of around 310 nm. The S_1_ state is well separated from the higher excited states. Upon excitation, the molecule is driven toward a shallow minimum characterized by an out-of-plane bending of the oxygen atom, see Figure 1b.

The subsequent photodecomposition mechanism involves two possible pathways, as summarized in Figure 1a. In the first pathway, extensively discussed in the literature,^58,62,77−79^ the ring strain is lifted by breaking one of the C–(C=O) bonds and subsequently releasing the CO fragment. This almost barrierless path is funneled by a conical intersection with a half-open biradical structure (CI1 in Figure 1b). The second pathway decreases the ring strain by breaking the C–C bond opposite to the carbonyl group, resulting in an open biradical structure. It is funneled by another conical intersection, structurally resembling the biradical product (CI2 in Figure 1b). The appearance of this pathway is scarce, since the potential energy surface drives the molecule toward the CO release. Therefore, this pathway has never been observed in simulations, although it was proposed experimentally as a cause of polymerization on the walls of the reaction vessel.^78^ Hence, it contributes significantly to the product formation due to the polymerization radical chain reaction. As we show later, our comparatively large sample combined with the statistical analysis allowed for the first observation of this channel in the dynamical calculations. For both channels, the recovery of cyclopropanone in the ground state (photostable channel) is also possible.

Both reactions have analogous pathways in the ground state, yet they are subject to activation barriers (dashed lines in Figure 1b). The transition states (TS1 and TS2) are reminiscent of their respective conical intersections and are energetically approximately 0.5 eV below them. The barrier heights are well above the thermal energy, although both products should be accessible in the hot ground state after excitation. In such cases, the polymerization pathway should be preferential.

### Electronic Structure Methods and Potential Energy Surfaces

#### Benchmark of Methods

**Figure 2:**
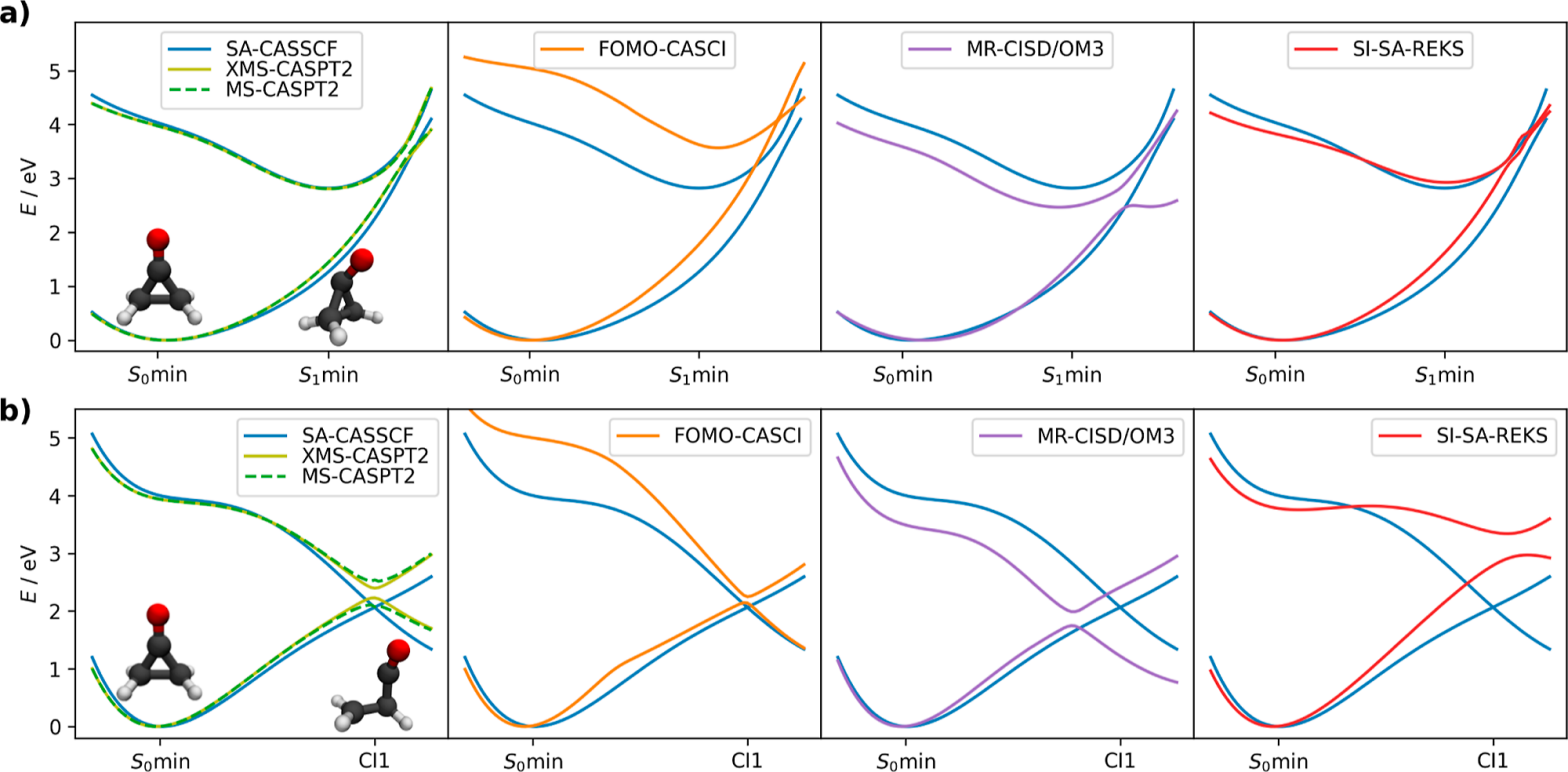
Potential energy surfaces at different levels interpolated between (a) the S_0_ and S_1_ minima and (b) the S_0_ minimum and the conical intersection CI1. The minima and conical intersection were optimized at the SA-CASSCF level.

The selection of the electronic structure level used for coupled electron–nuclear dynamics simulations is typically based on the inspection of important points on the potential energy surfaces. Here, we present two different cuts through the potential energy surfaces that are representative of the photodynamics: (a) interpolation between the S_0_ and S_1_ minimum which represents the out-of-plane oxygen bending upon the excitation (see Figure 2a,b) interpolation between the S_0_ minimum and conical intersection CI1 which is close to the reaction coordinate in the excited state (see Figure 2b). Such a reaction coordinate is typically used in photodynamical studies to benchmark electronic structure. We used the same geometries for all of the methods with minima and conical intersections optimized at the SA-CASSCF(8,7) level.

We observe that all of the methods yield a similar qualitative picture, differing just by an energy offset. The CASSCF and CASPT2 methods agree even quantitatively. The energy offsets observed for the FOMO-CASCI and MR-CISD/OM3 methods are not surprising as both are based on serious approximations: the FOMO orbitals are not fully optimized for the excited states, hence the shift to higher energies, and the OM3 Hamiltonian was not parametrized specifically for the cyclopropanone molecule. The only noticeable exception is the SI-SA-REKS method, which does not exhibit a steep slope along the second interpolation coordinate driving the molecule toward the open-ring structure of CI1 unlike the others, yet this might be biased due to the conical intersection optimization at the SA-CASSCF level. Also, the SI-SA-REKS potential energy curves exhibit spurious energy crossings between the electronic states behind the S_1_ minima, unlike the other methods. Thus, comparable photodynamics could be expected for all of the methods except SI-SA-REKS based on the inspection of potential energy surfaces.

#### Conical Intersections with Dynamical Correlation

As the population transfer occurs in the vicinity of conical intersections, we also took a close look at the CI1 conical intersection. This is especially interesting for the CASPT2 family of methods, which has a long history of developments in the context of the true degeneracy points. Although it is now agreed that the XMS-CASPT2 approach solves the problem of smoothness around the intersection seam,^80^ MS-CASPT2 was widely applied for nonadiabatic dynamics of many molecules^81−84^ and it is of interest to estimate an error associated with this choice.

**Figure 3:**
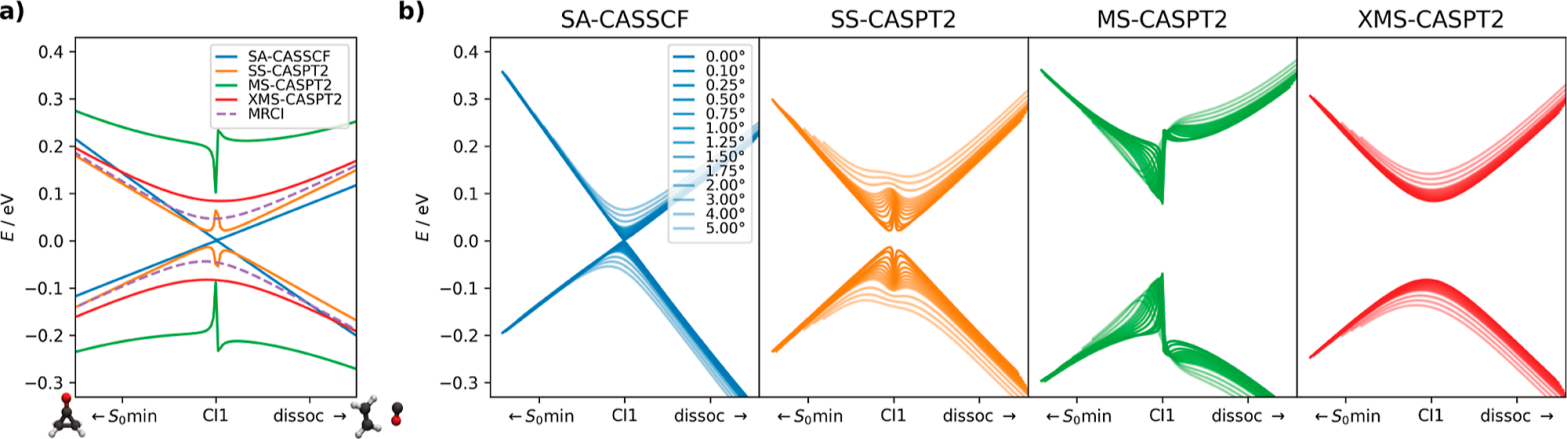
(a) Potential energies in the vicinity of CI1 calculated with the SA-CASSCF, SS-CASPT2, MS-CASPT2, XMS-CASPT2, and MRCI methods. (b) Series of scans of the potential energy surface passing around CI1 calculated with the SA-CASSCF, SS-CASPT2, MS-CASPT2, and XMS-CASPT2 methods. The more transparent the lines are, the further from CI1 the scan passes. The deviation from the exact CI in the dihedral angle of oxygen ranges from 0 to 5° at the reference SA-CASSCF level.

Therefore, we zoomed the potential energy surface from Figure 2b and performed a very fine scan of potential energies; see Figure 3a. The scan spans a small range of 0.08 Å of the C–C bond length, 2° of the C–C=O and 5.1° of the C–C–C=O dihedral angle. The MRCI calculation is presented for comparison as a reliable reference. From all the CASPT2 flavors, only the XMS-CASPT2 yields smooth physical curves with the splitting of the states slightly larger than for MRCI. Both SS-CASPT2 and MS-CASPT2 diverge at the exact SA-CASSCF conical intersection. This behavior is well documented in the literature.^85−89^ Interestingly, the divergence is larger for MS-CASPT2 rather than SS-CASPT2 although MS-CASPT2 is believed to be an improvement of the SS-CASPT2 formalism.^90^ Moreover, MS-CASPT2 predicts the largest splitting of the states and lies the furthest from MRCI.

Nevertheless, hitting the exact crossing of the potential energy surfaces is rarely the case in dynamics. Trajectories typically do not hit the crossing point exactly but evolve only in its vicinity.^91^ Therefore, we also inspected how quickly the divergent behavior disappears when moving away from the exact crossing; see Figure 3b. The observed divergence is quickly removed for SS-CASPT2 and the surfaces become smooth. Contrarily, the divergent behavior remains present in extended areas for MS-CASPT2. From this perspective, applying SS-CASPT2 appears more justified than MS-CASPT2, however unintuitive it sounds. Nonetheless, only the dynamical simulations can reveal how much is this translated into the observed lifetimes and quantum yields.

An extended discussion including the NEVPT2 and MC-PDFT methods is available in the Supporting Information.

### Nonadiabatic Dynamics

**Figure 4:**
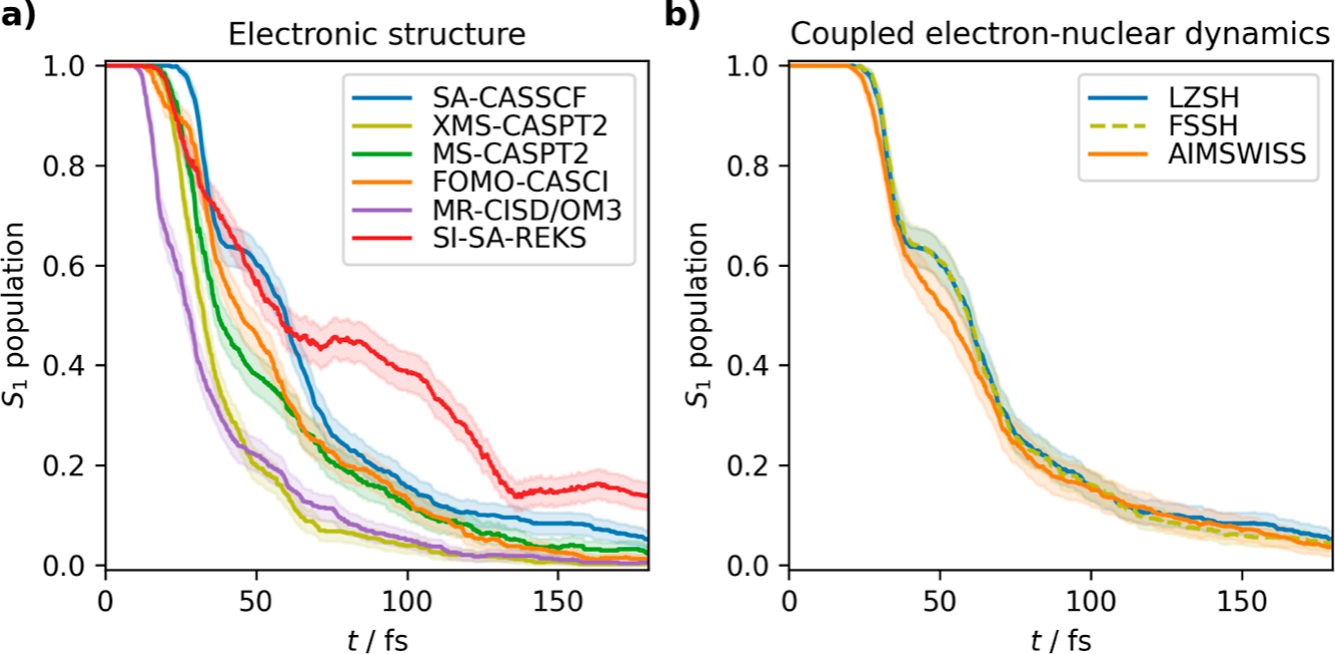
Populations of the S_1_ state for (a) different electronic structure methods with LZSH and (b) different methods for coupled electron–nuclear evolution with the SA-CASSCF electronic structure.

#### Excited-State Populations

The first compared property is the excited-state lifetime; see Figure 4. At first sight, it is immediately obvious that the variability is much bigger for the electronic structure methods as compared to the different algorithms of coupled electron–nuclear dynamics. The lifetimes are evaluated in Table 1. Within the *ab initio* family, the lifetimes range from 39 fs for the fastest XMS-CASPT2 simulation to 68 fs for the slowest SA-CASSCF simulation. The MS-CASPT2 with 54 fs and FOMO-CASCI with 58 fs appear between them. The difference in lifetimes of almost factor 2 between SA-CASSCF and XMS-CASPT2 is somewhat surprising when taking into account negligible differences in the potential energy surfaces. Focusing on all methods, semiempirical MR-CISD/OM3 is the fastest with a lifetime of 36 fs as it starts the population transfer sooner than the other methods. Its lifetime is within the error bars of the XMS-CASPT2 method. Contrarily, density-functional SI-SA-REKS is an outlier with the longest lifetime of 90 fs and different qualitative behavior as the population transfer exhibits a sudden stop at 140 fs and keeps constant. This is consistent with the REKS population trace calculated previously by Filatov et al.^58^ Note that recent ΔSCF calculations yielded even longer excited-state lifetime 137.0 ± 0.13 fs.^49^

Unlike electronic structure methods, the different coupled electron–nuclear algorithms are consistent in population transfer, especially in the case of FSSH and LZSH. This points out that with well-defined conical intersections, the transition formulas are equivalent, and we can save computer time by using the cost-effective LZSH. A small difference is observed for the AIMSWISS population but it is still kept within the error bars. The difference is possibly due to a more continuous spawning procedure inherent to AIMSWISS which makes the population transfer more gradual and smears out the stepwise structure of the TSH results.

Details about fitting the populations and lifetime calculations are provided in Supporting Information.

#### Quantum Yields of CO Release and Polymerization

The quantum yields of the CO release are presented in Table 1. Again, a larger variability of data can be seen for electronic structure methods. For the *ab initio* methods, the quantum yields are consistent within the error bars and very close to the experimental value of 0.73 (average weighted by absorption intensity).^77^ The semiempirical MR-CISD/OM3 method, which exhibited a very promising population transfer comparable to XMS-CASPT2, overestimates the quantum yield to 0.89, getting further from the experimental value. The density-functional SI-SA-REKS method fails completely, with a very low quantum yield of 0.20. This value is strikingly different from those of the other methods and also the experiment. However, we note that SI-SA-REKS simulations of Filatov et al.^58^ yielded higher quantum yields of 0.44 ± 0.11 which do not match our results. We made several attempts to reproduce the published results with no success. Still, the 0.44 quantum yield of Filatov et al. is far from the other methods and mainly the experiment. Hints of problems for SI-SA-REKS were already present during the inspection of potential energy surfaces, though now they are confirmed by the slower population transfer and extremely low quantum yield.

As for the coupled electron–nuclear algorithms, their quantum yields are identical within statistical error. Again, this confirms a low sensitivity to coupled electron–nuclear dynamics compared to the electronic structure.

We have also compared the appearance of the polymerization photochemical pathway; see Table 1. Discovering and quantifying the polymerization pathway was largely facilitated and simplified by the MDS procedure, which allows for fast identification of even small clusters in large data sets; see the Supporting Information. Experimentally, this pathway was robustly observed although it is not possible to establish its quantum yield.^78^ Therefore, this comparison is rather binary, whether the channel is or is not present. As for the electronic structure, we see that all *ab initio* methods predict polymerization pathways at least once while the DFT and semiempirical ones do not. For the coupled electron–nuclear algorithms, we consistently observe this pathway for all of the techniques. Thus, we again see larger discrepancies within the electronic structure than those for the nuclear evolution. However, the rare-event character would require an even larger statistical sample to draw determining conclusions.

#### Transition Region Analysis

Regions of strong nonadiabatic couplings, where the molecule transfers (hops or spawns) between electronic states, often determine the outcome of the dynamics. Typically, the minimum energy conical intersection would be compared for the electronic structure methods to deduce their behavior. However, the dynamics may not drive the molecule to the minimum energy conical intersection and transfer further away.^91^ As for the coupled electron–nuclear algorithm, they might follow the same path but choose a different place to hop/spawn. Thus, comparing the transition geometries for the dynamics should reveal important information about the methods and provide more insight into their comparison.

**Figure 5:**
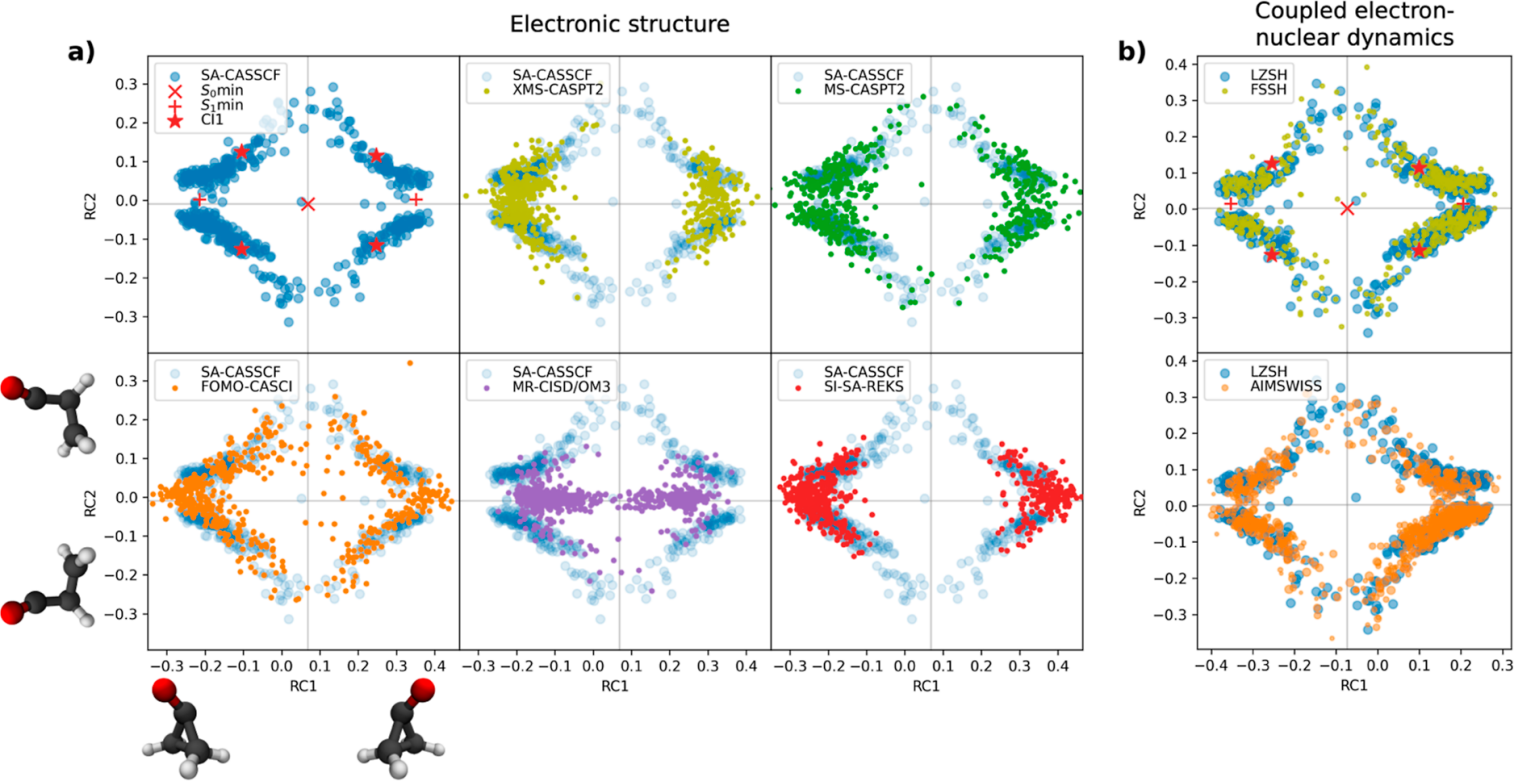
Hopping/spawning geometries of different (a) electronic structure methods and (b) coupled electron–nuclear algorithms represented in two reduced dimensions by MDS. RC1 corresponds to oxygen out-of-plane bending, while RC2 corresponds to ring-opening next to the carbonyl group.

The representations of the hopping/spawning geometries in two dimensions generated by MDS are presented in Figure 5. Initially, the geometries were aligned according to all carbon atoms. The distance matrix was calculated from the carbon and oxygen atoms. The hydrogen atoms were excluded since they bring no mechanistic information but only increase the noise of the data. The first reduced coordinate (RC1) corresponds to the out-of-plane bending of oxygen, while the second reduced coordinate (RC2) corresponds to ring-opening next to the carbonyl group. Both these coordinates are symmetric; thus, two axes of symmetry emphasized by gray lines are present in this plot: the vertical axis reflects the oxygen out-of-plane bending symmetry, and the horizontal one reflects the ring-opening symmetry. To guide the reader’s eye, the S_0_min, S_1_min, and CI1 are also marked in the diagram. The movement of the wave packet after excitation corresponds dominantly to the out-of-plane oxygen bonding, i.e., the wave packet goes from the S_0_ minimum toward S_1_ minimum. Then, the ring opens and the wave packet heads toward CI1, where it starts transferring to the ground state.

**Table 1:** Lifetimes of the Excited State (τ), Quantum Yields of Photodissociation (ϕ_CO_), and Counts of the Polymerization Pathway (*N*_polymer_) for Different Methods of Electronic Structure and Coupled Electron–Nuclear Evolution

	method	τ/fs	ϕ_CO_	*N*_polymer_
electronic structure	SA-CASSCF(8,7)	67.7 ± 4.6	0.76 ± 0.04	4
	XMS-CASPT2(8,7)	39.1 ± 3.1	0.78 ± 0.03	1
	MS-CASPT2(8,7)	53.9 ± 4.2	0.76 ± 0.03	2
	FOMO-CASCI(8,7)	57.6 ± 4.3	0.73 ± 0.04	7
	MR-CISD/OM3(8,7)	36.1 ± 3.5	0.89 ± 0.03	0
	SI-SA-REKS(2,2)	90.3 ± 6.9	0.20 ± 0.03	0
coupled el.-nuc. dynamics	LZSH	67.7 ± 4.6	0.76 ± 0.04	4
	FSSH	66.7 ± 4.5	0.76 ± 0.03	7
	AIMSWISS	63.4 ± 4.5	0.75 ± 0.04	1
experiment^a^			0.73	observed

a The experimental quantum yield was taken from ref (77) as an average throughout the spectrum weighted by the absorption intensity.

Let us start with the differences between the electronic structure methods. The SA-CASSCF result is taken as a reference for comparison with other methods. A characteristic feature of the SA-CASSCF hopping geometries is that the ring is always open, leaving the region around the horizontal axis of symmetry empty (horizontal gray line). This feature disappears for all of the other methods which have a quite dense population around the horizontal axis of symmetry. We can also deduce some information about the shape of the intersection seam—the more the ring is open, the less is the oxygen bent out-of-plane and vice versa. The FOMO-CASCI method has the distribution of hopping geometries most similar to SA-CASSCF except that it fills the empty region around the horizontal axis of symmetry corresponding to a closed ring structure. This explains why FOMO-CASCI predicts a faster population transfer. Since the molecule first bends the oxygen moving toward S_1_ minimum (along the horizontal axis) and then the ring opens (moving vertically), the hops with a closed structure occur prior to the ones with an open ring. With the same reasoning, we explain why CASPT2 methods are even faster: their distributions are denser close to the S_1_ minimum. The distribution produced by MR-CISD/OM3 is focused dominantly around the horizontal axis of symmetry closer to the S_0_ minimum. The hopping structures rarely open the ring. This rationalizes why the method provides the fastest population transfer. Interestingly, the SI-SA-REKS distribution is very similar to the XMS-CASPT2 one. This is rather surprising, as SI-SA-REKS lifetimes and populations do not compare with those of XMS-CASPT2. Thus, the shapes of potential energy surfaces are qualitatively different in these regions.

#### SI-SA-REKS: the Effect of Active Space

The failure of the SI-SA-REKS(2,2) method can possibly stem from the small active space used rather than being an inherent failure of the method. We tested the effect of the smaller active space at the SA-CASSCF(2,2)/LZSH level, comparing the data with the SA-CASSCF(8,7) and SI-SA-REKS(2,2) results. The smaller active space prolongs the lifetime of SA-CASSCF dynamics from 67.7 ± 4.6 for (8,7) up to 92.7 ± 6.7 fs, which matches the 90.3 ± 6.9 fs of SI-SA-REKS. The quantum yield of CO release is also shifted to a lower value of 0.52 ± 0.04 closer to the SI-SA-REKS value of 0.20 ± 0.03. The polymerization pathway was not spotted for SA-CASSCF(2,2) analogically to SI-SA-REKS and their hopping geometries overlap nicely. To conclude, while the decrease in active space can explain the longer lifetimes and absence of the polymerization pathway for SI-SA-REKS, it can only partially account for the very low quantum yield. Hence, other aspects of the SI-SA-REKS method probably also play a role. With the limitation of a small active space, SI-SA-REKS cannot be a general method for nonadiabatic dynamics, and further development in terms of an arbitrary active space is necessary. For more details on the active space size benchmark, see Supporting Information.

### CASPT2 Variants in Nonadiabatic Dynamics

**Figure 6:**
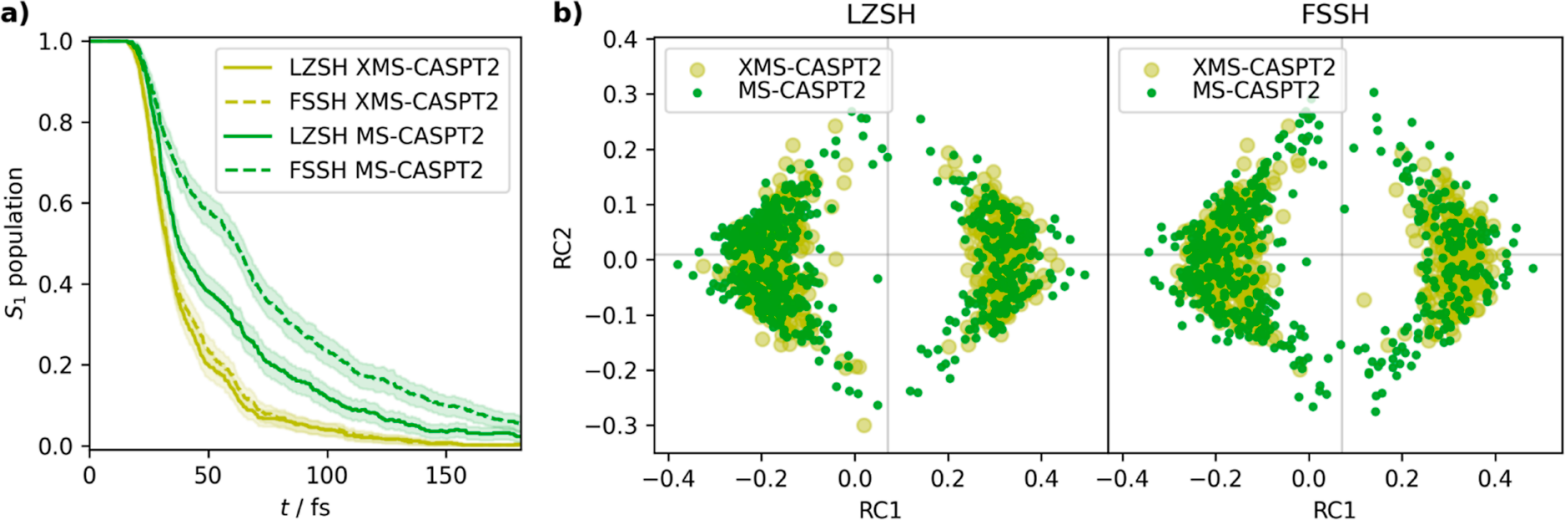
Comparison of XMS-CASPT2 and MS-CASPT2 nonadiabatic dynamics with LZSH and FSSH. (a) Populations of the S_1_ state. (b) Hopping geometries represented by MDS in two reduced dimensions. RC1 corresponds to oxygen out-of-plane bending while RC2 corresponds to ring-opening next to the carbonyl group.

In one of the previous sections, we investigated the divergent behavior of MS-CASPT2 close to the conical intersection. We also showed that there is a discrepancy between MS- and XMS-CASPT2 lifetimes at the LZSH level of theory. To obtain more insight into the effects of conical intersection description in nonadiabatic dynamics, we also performed FSSH simulations for both MS- and XMS-CASPT2 and analyzed them together with LZSH, see Figure 6. Focusing on the excited-state populations, we observe a large sensitivity on coupled electron–nuclear algorithms for MS-CASPT2 while there is only a negligible difference for a well-behaved XMS-CASPT2 electronic structure. These observations are in line with previous experience with LZSH. The LZSH method is simple to use, yet it is less forgiving with respect to potential energy surface inaccuracies. On the other hand, there is no profound difference between hopping geometries distributions. Also, the quantum yields for XMS-CASPT2 (0.76 ± 0.03) and MS-CASPT2 (0.79 ± 0.04) with FSSH are in line with the LZSH results of other *ab initio* methods and also experiment presented in Table 1. More details on the comparison are provided in Supporting Information.

## Discussion

The main message of this study is that it is the electronic structure theory that chiefly controls the outcome of the ultrafast nonadiabatic simulations in excited states. The algorithm used for the coupled electron–nuclear evolution is of secondary importance in the present case of the cyclopropanone molecule. We note that our conclusion has its limitations as there are other parameters that might affect the dynamics such as the role of initial conditions,^92^ decoherence corrections,^93^ velocity rescaling,^94^ or the nonadiabatic couplings treatment.^95^ More importantly, our conclusion is undoubtedly system-dependent. Certain differences between various coupled electron–nuclear evolution algorithms have been encountered for a number of molecules. Nevertheless, looking at the study on molecular Tully’s models probing typical types of nonadiabatic transitions by Ibele and Curchod, profound discrepancies were found only for the fulvene molecule (Tully model III).^93^ The AISM and TSH results were in good agreement for ethylene (Tully I) and DMABN (Tully II). Furthermore, the community is well aware of different coupled electron–nuclear algorithm limitations as an abundance of benchmarks is available. On the other hand, electronic structure benchmarks in nonadiabatic dynamics are scarce, although inferring the effects of electronic structure from inspecting a few points on the potential energy surface might be misleading, the small eight-atom cyclopropanone molecule being a good example. We argue that observing the strong effect of electronic structure on such a simple molecule, which appears to be insensitive to coupled electron–nuclear algorithms, only emphasizes the crucial role electronic structure plays in nonadiabatic dynamics.

Let us put these findings in the context of the current state of computational photodynamics. We usually start with the mapping of the potential energy surfaces, i.e., with calculating energetics in the S_0_ minimum, locating the excited-state minima, minimum energy conical intersections, minimum energy crossing points with states of other multiplicities, transition states, and energetics of the final reaction channels. At this point, we typically can afford high-quality electronic structure theory to benchmark the affordable approaches. The method to be used for nonadiabatic dynamics is then chosen based on the benchmark and computational efficiency. However, our results indicate that a favorable comparison in geometries and energetics does not guarantee the same course of dynamics. Such benchmarks can discard obvious outliers, such as SI-SA-REKS in our case, but cannot provide decisive information. An example from our work is the SA-CASSCF and XMS-CASPT2 methods. Although their potential energy surfaces are quantitatively comparable on our scans, their lifetimes differ by a factor of almost 2. Another example is the MS- and XMS-CASPT2 methods that differ solely at the crossing point, yet their lifetimes are again significantly different.

This leads us to a more general conclusion as the present findings reopen the question of whether the common practice of photodynamics simulations should not be changed. In quantum chemistry, it is essentially unthinkable to publish a study without exploring the sensitivity of the results to different methods and basis sets. This gives some idea, albeit vague, of the systematic error of the calculated data. Such an approach is difficult in computational photodynamics due to the high cost of the simulations. One possible direction can be connected with the recent development of nonadiabatic simulations using machine learning-based potentials.^96−98^ Here, the sensitivity analysis should be relatively straightforward.

The main conclusion made above can be viewed as trivial. The decisive role of potential energy surface quality on the observed quantities is well appreciated in the ground state chemistry, and the photodynamics community is aware of the problem on a qualitative level too. Yet surprisingly few studies focus on this major problem in the field, and the sensitivity of our predictions to electronic structure are rarely discussed. This is, however, essential if we aim to reliably interpret experiments or predict new phenomena in nature.

Another topic that we have touched on is the well-known problem of perturbation theories and conical intersections. The MS-CASPT2 method, once frequently used, was replaced by its successor, XMS-CASPT2, which is able to properly deal with degeneracies. In our work, we have found divergences at a conical intersection and in its vicinity for MS-CASPT2. Nevertheless, it appears that even such spurious behavior does not invalidate the outcome of simulations completely. The quantum yields and distributions of hopping geometries were found to be almost the same for MS- and XMS-CASPT2. A pronounced distinction was found only for the excited-state lifetime, differing by a factor of 1.4 for LZSH and 1.8 for FSSH. These results bring a sense of reliability for previous MS-CASPT2 simulations, although XMS-CASPT2 should be used preferentially for the photodynamical simulations.

We have also shown that the similarity of different approaches (being it either electronic structure levels or nuclear dynamics algorithms) cannot be judged based on a single quantity, e.g., the time evolution of electronic populations. The MR-CISD/OM3 method is a prominent example in our study. Its population trace nicely matches the XMS-CASPT2 population and appears as a very promising method. Nonetheless, the quantum yields of CO release significantly differ, and the hopping geometries barely overlap. Hence, only a set of quantities can reveal problems. In general, results of photodynamical simulations are often underanalyzed and one should aim at comparing more quantities, ideally with experimentally available counterparts.

## Conclusions

In summary, we demonstrated the electronic structure as the quality-determining component of the nonadiabatic dynamics of cyclopropanone. The calculated lifetimes and quantum yields appear to be highly sensitive to electronic structure methods, even when inspection of their potential energy surfaces shows qualitative agreement. This leads us to question the current protocol of photodynamical simulations, where the electronic structure is typically benchmarked only on a few points in the configuration space. However, inferring the effects of electronic structure from such a benchmark is difficult and often misleading. Therefore, we prompt more sensitivity analysis for photodynamical studies, e.g., with machine learning potentials. Additionally, we probed the effect of different multistate versions of CASPT2 on the nonadiabatic dynamics. Although we see clear deviation in lifetimes, the MS- and XMS-CASPT2 methods yield qualitatively similar dynamics, demonstrating a weak effect of the spurious MS-CASPT2 treatment of potential energy crossings. In light of our results, we believe that the previous MS-CASPT2 results still bear reliable information.

## Supporting Information Available

Details about initial conditions, benchmarks of the mechanism with MRCI and XMS-CASPT2, description of conical intersections with MC-PDFT and NEVPT2, fitting of populations and lifetimes, application of multidimensional scaling as a tool for discovering rare geometries in large samples, and comparison of nonadiabatic dynamics with MS-CASPT2 and XMS-CASPT2 (PDF)
