# Supplementary material for: What Controls the Quality of Photodynamical Simulations? Electronic Structure Versus Nonadiabatic Algorithm
Source: J Chem Theory Comput. 2023 Nov 8;19(22):8273–84. doi: 10.1021/acs.jctc.3c00908 (PMC10688183; doi:10.1021/acs.jctc.3c00908)
Supplement: Supplementary file 1 — ct3c00908_si_001.pdf [file ct3c00908_si_001.pdf]

Supporting information

What controls the quality of photodynamical simulations? Electronic structure vs nonadiabatic algorithm

Jiří Janoš and Petr Slavíček*
University of Chemistry and Technology, 166 28 Prague 6, Czech Republic
* Corresponding author: Petr.Slavicek@vscht.cz

I. Active space orbitals

Throughout the work, we have used two active spaces: *i*) (8,7) composed of two σ_{CC}, π_{CO}, n_{O}, π^*_{CO} and two σ^*_{CC} orbitals for SA-CASSCF, XMS-CASPT2, MS-CASPT2, FOMO-CASCI and MR-CISD/OM3, and ii) (2,2) composed of only the n_{O} and π^*_{CO} orbitals for the SI-SA-REKS and also one set of SA-CASSCF simulations presented in SI, see **Figure S1**.

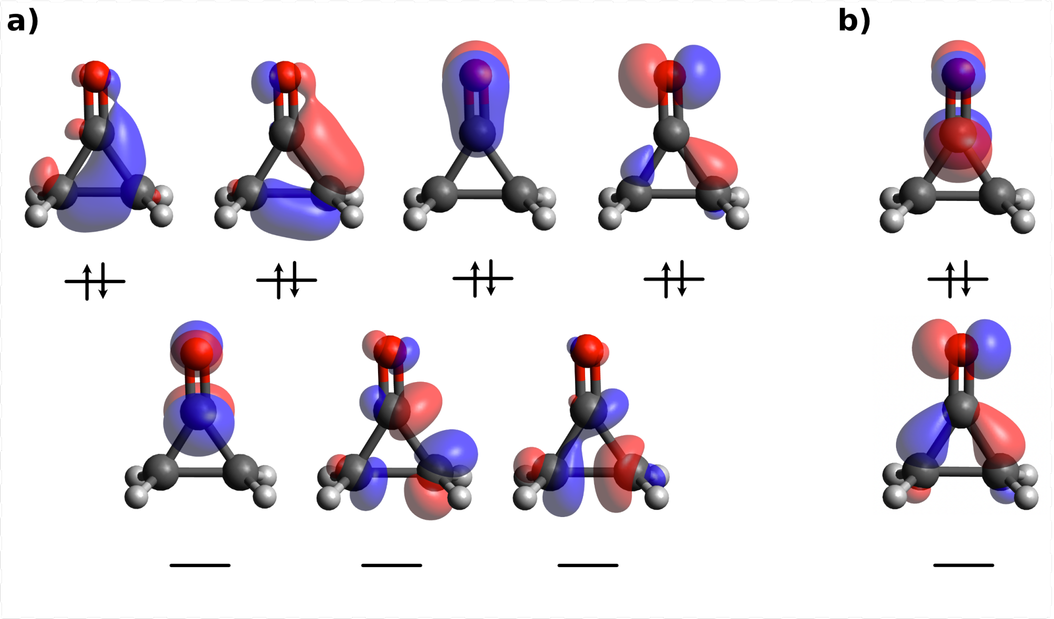

Figure S1. Orbitals composing the a) the (8,7) active space (calculated with SA-CASSCF) and b) the (2,2) active space (calculated with SI-SA-REKS).

II. Initial conditions

The initial conditions were sampled with the ground-state molecular dynamics with constrained excitation wavelength.[1] This approach mimics the continuous-laser experiment with the excitation energy precisely defined. Thus, only geometries with the corresponding excitation energy are promoted to the excited states. We use this approach to efficiently sample selected regions of the spectra, e.g. the spectral tails. For cyclopropanone, the tails are especially interesting, since different

quantum yields with respect to the center of the peak were measured in both the high and low photon energies.[2, 3] Therefore, we wanted to emphasize also this region in our simulations as a nonstandard behavior might occur there.

The ground state was sampled with SA-CASSCF(2,4) method. We selected six excitation wavelengths scattered across the whole spectrum: 261 nm, 272 nm, 298 nm, 330 nm, 354 nm and 400 nm. The 400 nm pulse is in a very low-intensity region almost out of the spectrum because the experiment was measured also in these parts. The positions of the sampling pulses with respect to the calculated and measured spectra are shown in **Figure S2a**. These initial conditions were then used for all the nonadiabatic dynamics with different electronic structure methods. We note that when switching to a different electronic structure method, the excitation wavelength is blurred into narrow peaks, see **Figure S2b**. This would correspond to a peak with a finite duration.

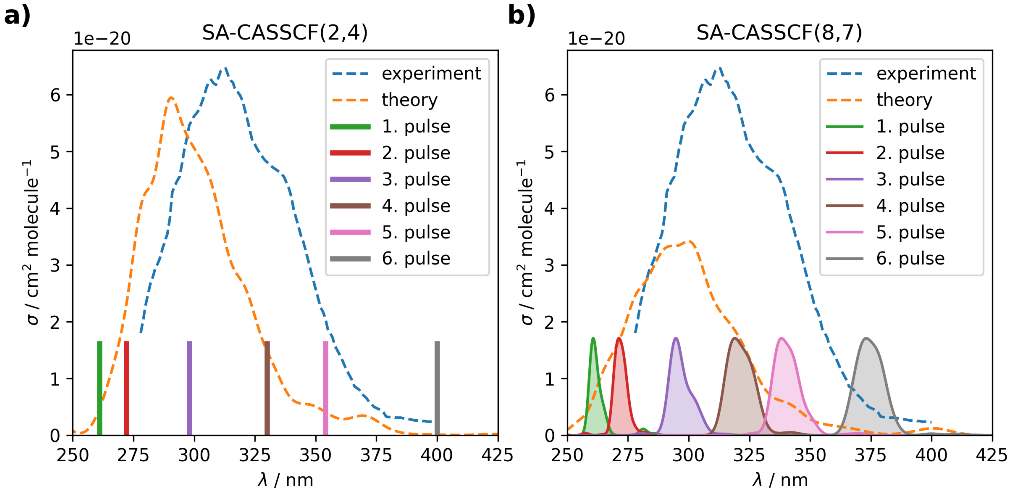

Figure S2. Absorption spectra and excitation pulses at the a) SA-CASSCF(2,4) and b) SA-CASSCF(8,7) level of theory.

Since we used the same set of initial conditions for all nonadiabatic simulations, the excitation energies for the applied electronic structure methods differ. Thus, we also report the normalized spectra of our pulses for all the methods, see **Figure S3**. The trends are in line with the potential energy surfaces presented in the main article: SA-CASSCF, XMS-CASPT2, MS-CASPT2 and SI-SA-REKS overlap while FOMO-CASCI is shifted towards higher energies and MR-CISD/OM3 is shifted to lower energies.

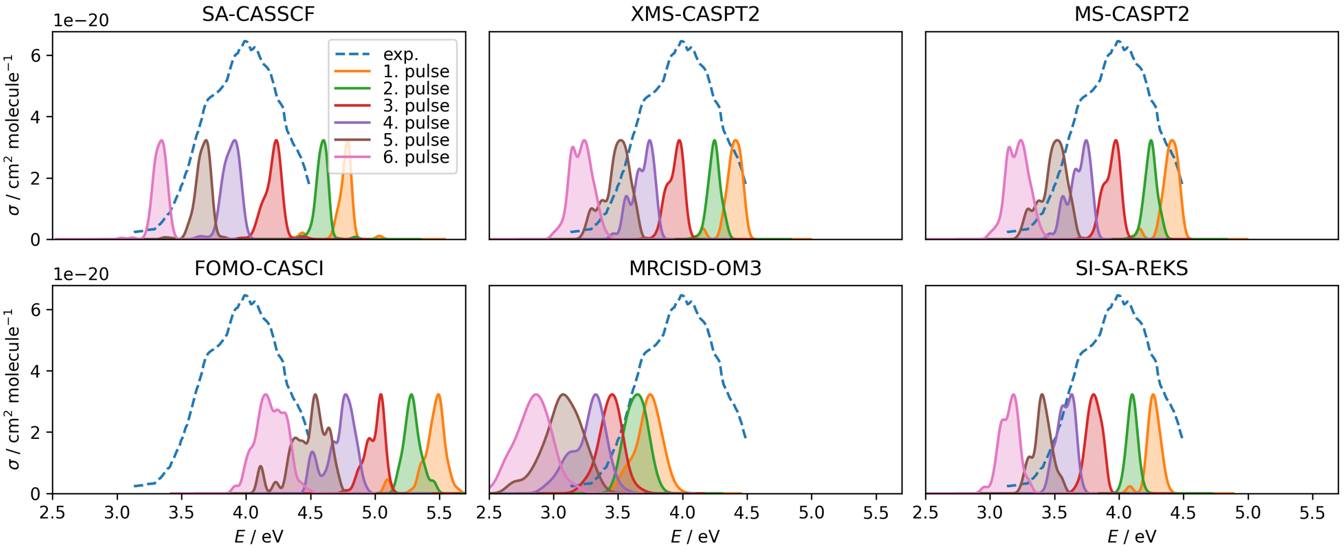

Figure S3. The normalized spectra of the excitation pulses with different electronic structure methods.

To get more insight into the initial conditions, we also compared their overlap using the MDS algorithm described in the main article. The distributions corresponding to the six pulses were processed together with the ground-state distribution sampled with molecular dynamics, see **Figure S4**. The first and third reduced coordinates (RC1 and RC3) were plotted as they contained the largest variance between the data sets. RC1 can be attributed to the out-of-plane bending of the oxygen atom while RC3 corresponds to the C=O bond stretching. We see that the first pulse lies on the edge of the ground-state distribution with a short C=O bond length and oxygen in the plane of the carbon ring. With higher wavelengths, the pulses move up in RC3 prolonging the C=O bond with the third pulse centered around the S_0 minimum and the fourth with an even longer bond length. For the first three pulses, the dihedral angle is centered around its zero position. With the fourth pulse, the distribution of the dihedral angle becomes wider as the oxygen gets more flexible. In the last two pulses, the oxygen atom slipped on one side of the carbon ring and was no longer able to reverse on the other side resulting in the distribution positioned only on one side instead of being symmetric around the planar geometry. With higher wavelengths, the pulses get closer to the S_1 minimum which has an even longer C=O bond and the oxygen atom is in an out-of-plane position.

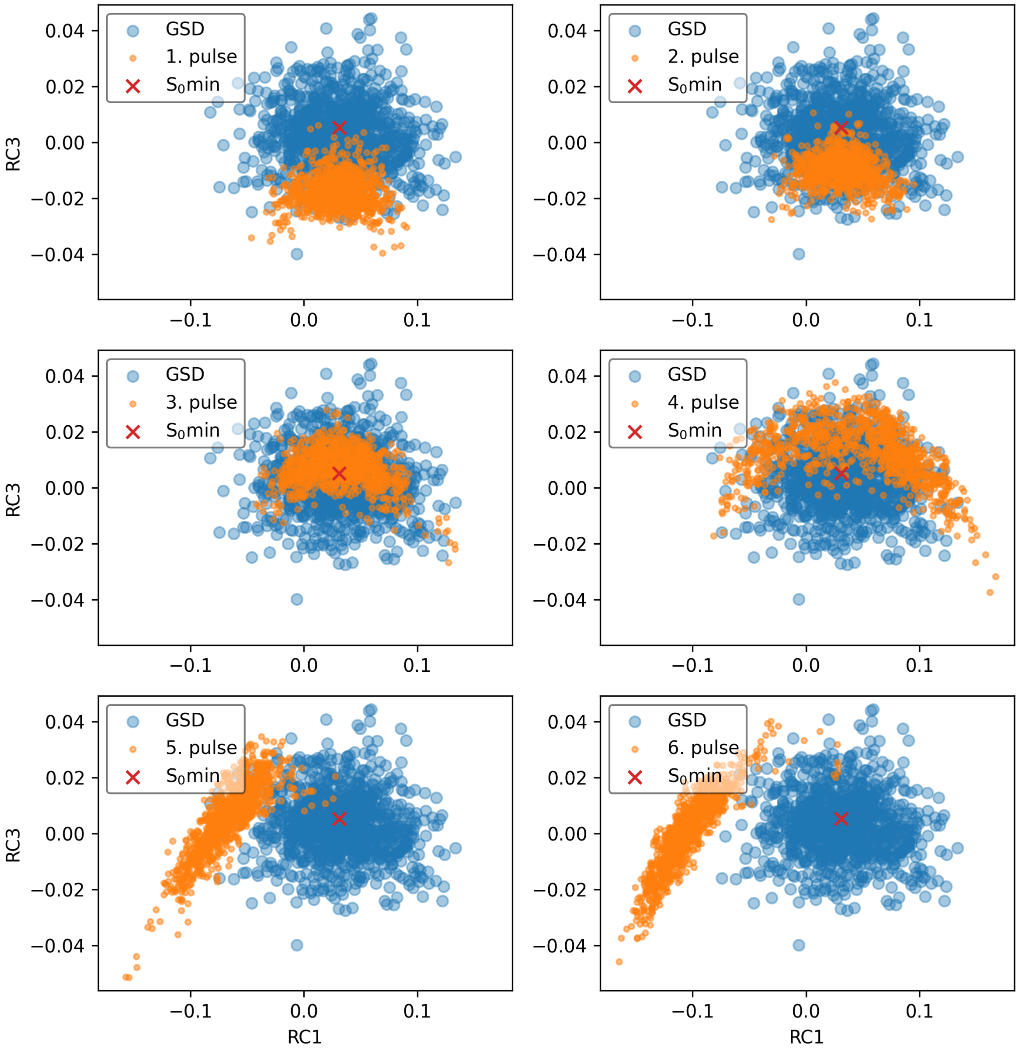

Figure S4. A thousand randomly selected geometries from the sampling of the pulses are plotted against the ground-state distribution (GSD) in the first and third reduced coordinates produced by MDS. RC1 represents the C=O bond length with negative values corresponding to shorter bond length and positive to longer. RC3 represents C–C–C=O dihedral angle, a value of 0.03 corresponds to zero dihedral angle.

At the end of the discussion about initial conditions, we would like to note that the starting conditions could introduce a bias toward a given electronic structure method. However, we do not expect the bias to be of crucial importance as all the methods exhibit similar behaviour around the S_0 minima.

III. Mechanism of photochemical decomposition with MRCI and XMS-CASPT2

To get a better view of the energetics presented in **Figure 1**, we recalculated the energies of the CASSCF structures with the MRCI and XMS-CASPT2 methods, see **Table S1**. Both these methods recover the dynamical correlation which is especially important for dissociation energies as fewer electrons are correlated after bond dissociation. The excitation energy and S_1 minimum energy are barely affected while both conical intersections are shifted to higher energies. This effect of dynamical correlation is larger for CI2 and puts it on the same energy as CI1. Placing a conical intersection higher in energy could result in smaller kinetic energy during the non-adiabatic transition influencing the transition probabilities. Yet, the biggest change can be seen in both products and transition states. While P2, TS1, and TS2 are shifted to higher energies because biradicals with two single uncorrelated electrons are formed, the P1 structure gets lower in energy as the CO release creates two closed-shell molecules. This makes the ground-state reactions less probable and the CO release even more favorable over the polymerization. The effect of dynamical correlation is always stronger for XMS-CASPT2 than for MRCI.

Table S1. Recalculated energies of the SA-CASSCF(8,7) structures from **Figure 1** at the MRCI(8,7) and XMS-CASPT2(8,7) levels. Since the geometries of CI1 and CI2 were not reoptimized with these methods, the S_0 energy (the first number) and S_1 energy (the number in parentheses) are not equal.

structure	SA-CASSCF / eV	MRCI / eV	XMS-CASPT2 / eV
S_0min	0.00	0.00	0.00
S_1	4.13	4.13	4.00
S_1min	2.79	2.90	2.80
CI1	2.01	2.21 (2.31)	2.22 (2.38)
CI2	1.57	2.11 (2.14)	2.22 (2.35)
P1	-0.53	-0.65	-0.76
P2	0.78	1.33	1.55
TS1	1.50	1.84	2.01
TS2	0.82	1.28	1.46

III.A Cartesian coordinates

We provide Cartesian coordinates of the geometries used in the main article and in the benchmark above. All geometries were optimized at the SA2-CASSCF(8,7)/6-31g* level of theory.

S_0min

$E_{\text{SA2-CASSCF(8,7)/6-31g*}}$ = -190.79379319 a.u.

C	-0.05048703	0.16280272	-0.18664043
C	0.34707434	0.26730414	1.36052158
C	1.29874618	-0.14264080	0.26622185
H	-0.69772377	-0.64895451	-0.47132165
H	-0.04381800	-0.48740015	2.02047496
H	0.43079845	1.25438543	1.78072134
H	-0.22448789	1.08713902	-0.71033960
O	2.40352983	-0.48471102	-0.03192189

S_1min

$E_{\text{SA2-CASSCF(8,7)/6-31g*}}$ = -190.69130831 a.u.

C	-0.03403527	-0.73133802	-0.99654559
C	-0.32419635	0.04174437	0.33899421
O	0.40003399	-0.05926571	1.37578592
C	0.15653199	0.73161411	-0.98711349
H	1.15942828	1.11856899	-0.96856405
H	-0.57618016	1.35905019	-1.46240595
H	-0.90222745	-1.14403074	-1.47859698
H	0.83623897	-1.36249619	-0.98459308

CI1

$E_{\text{SA2-CASSCF(8,7)/6-31g*}}$ = -190.71985011 a.u.

C	0.04979549	-0.66143588	-0.98166683
C	0.15344876	0.82664613	-1.04910743
C	-0.03642769	0.96567649	0.43518755
H	-0.92196722	-1.11302189	-0.93357146
H	-0.63742766	1.30870476	-1.60470855
H	1.11687984	1.21196431	-1.35807744
H	0.89050157	-1.22429913	-0.62538366
O	0.44096991	1.07448921	1.51424182

CI2

$E_{\text{SA2-CASSCF(8,7)/6-31g*}}$ = -190.73534124 a.u.

C	-1.34203053	0.02320454	-0.51707421
C	-0.02999431	-0.13800587	-0.12988447
O	0.19686736	-0.48451736	1.19011599
C	1.10593778	0.01284947	-0.95318119
H	2.09562453	-0.10312841	-0.56267928
H	0.98236633	0.26995407	-1.98642896
H	-2.14940740	-0.15086725	0.16852807
H	-1.57312677	0.31411380	-1.52406095

TS1

$E_{\text{SA2-CASSCF(8,7)/6-31g*}}$ = -190.73878889 a.u.

C	-0.66655752	0.08066372	1.58611355
C	0.63321634	0.15703833	0.77349041
C	-0.02051615	-0.14112209	-0.53357814
O	0.23445474	-0.06729155	-1.68724044
H	-1.42457144	0.80841643	1.35362192
H	-1.00193170	-0.87716777	1.93937486
H	1.34119402	-0.61582550	1.05148089
H	1.13149172	1.12029743	0.82645896

TS2

$E_{\text{SA2-CASSCF(8,7)/6-31g*}}$ = -190.76371365 a.u.

C	-1.26950383	-0.04509122	-0.55027437
C	0.02086337	0.09457894	0.09863910
O	0.23047023	0.08968315	1.31351495
C	1.01915314	-0.04534693	-0.94514777
H	1.85858345	-0.70028903	-0.81097298
H	0.94958309	0.52483310	-1.85088362
H	-2.01558577	-0.69984941	-0.14254210
H	-1.50732669	0.52508441	-1.42699821

P1

$E_{\text{SA2-CASSCF(8,7)/6-31g*}}$ = -190.81344757 a.u.

C	-0.00841160	-0.62599665	5.89813974
H	0.88914169	-1.21341094	5.97216518
H	-0.92396879	-1.09849547	6.20588493
C	0.01300991	0.63606996	5.44333915
H	0.92858999	1.10856998	5.13563616
H	-0.88454087	1.22347393	5.36924950
C	-0.00554020	0.47553829	-5.24157924
O	-0.00116174	-0.37153257	-5.96975299

P2

$E_{\text{SA2-CASSCF(8,7)/6-31g*}}$ = -190.76500654 a.u.

C	-1.19296007	0.70404164	-0.97618667
C	-0.27985022	-0.17515662	1.14239176
C	-0.01902808	0.30816934	-0.20812144
H	-2.07414391	1.07011060	-0.48550059
H	-1.13208200	0.17025113	1.69532264
H	0.47916132	-0.73075566	1.65788822
H	-1.11610072	0.80343233	-2.04139774
O	1.12517669	0.35523925	-0.68175518

IV. Conical intersections with MC-PDFT and NEVPT2

The main article discusses the effect of different CASPT2 flavors on conical intersection CI1. Here, we extend this study by n-electron valence state perturbation theory (NEVPT2)[4] and multiconfigurational pair-density functional theory (MC-PDFT)[5]. Both these approaches, NEVPT2 and MC-PDFT, are of a similar character as CASPT2 and, therefore, also prone to instabilities near conical intersections as they do not end up with Hamiltonian diagonalization. While NEVPT2 is a perturbation theory only with different partitioning of the Hamiltonian than CASPT2, MC-PDFT uses density-functional approaches to add the missing dynamical correlation to the SA-CASSCF energy. Both NEVPT2 and MC-PDFT applied in this work are of a single-state character; thus, they should be compared to the SS-CASPT2. Such comparison can bring more insight into the behavior of different groups of methods around conical intersections. We note that multistate MC-PDFT methods have already been developed to alleviate problems around conical intersection.[6]

Specifically, we use the strongly contracted (SC) and partially contracted (PC) single-state (SS) versions of NEVPT2 as implemented in the Molpro 2012.1 code[7, 8]. For the MC-PDFT, we tested the ftrevPBE and ftBLYP methods implemented in the OpenMolcas v18.09 package [9, 10].

The scans of potential energy surfaces around the conical intersection show that both SS-NEVPT2 variants exhibit similar

divergencies that are comparable to SS-CASPT2, see **Figure S5**. This suggests that the problem is not sensitive to the partitioning of the Hamiltonian. Looking at MC-PDFT, we observe much larger discrepancies than for perturbation theories. At the exact crossing point of the SA-CASSCF reference, the S_0 and S_1 states switch ordering and then cross again further. Nevertheless, we need to emphasize again that multistate variants are available in newer versions of the code. These should be compared with MS- and XMS-CASPT2 as done in reference [6].

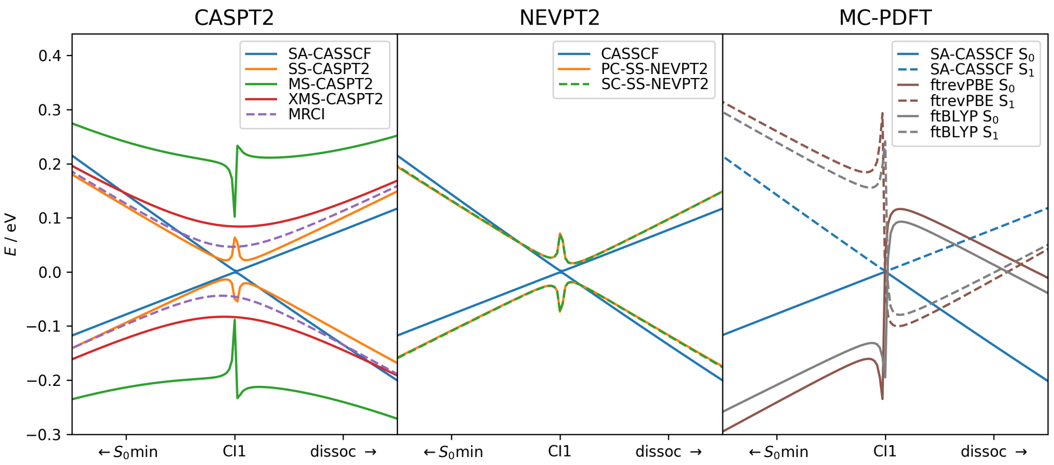

Figure S5. Potential energies in the vicinity of CI1 calculated with the SA-CASSCF, SS-CASPT2, MS-CASPT2, XMS-CASPT2, MRCI, PC-SS-NEVPT2, SC-SS-NEVPT2, ftrevPBE and ftBLYP methods.

Analogically to the main article, we also calculated the series of scans close to the CI1 to see how quickly the divergences disappear, see **Figure S6**. For both SS-NEVPT2 versions, we again see a very similar behavior as for SS-CASPT2. For the MC-PDFT ftrevPBE method, the problems do not disappear even far away from the conical intersection with the switching of the states still present.

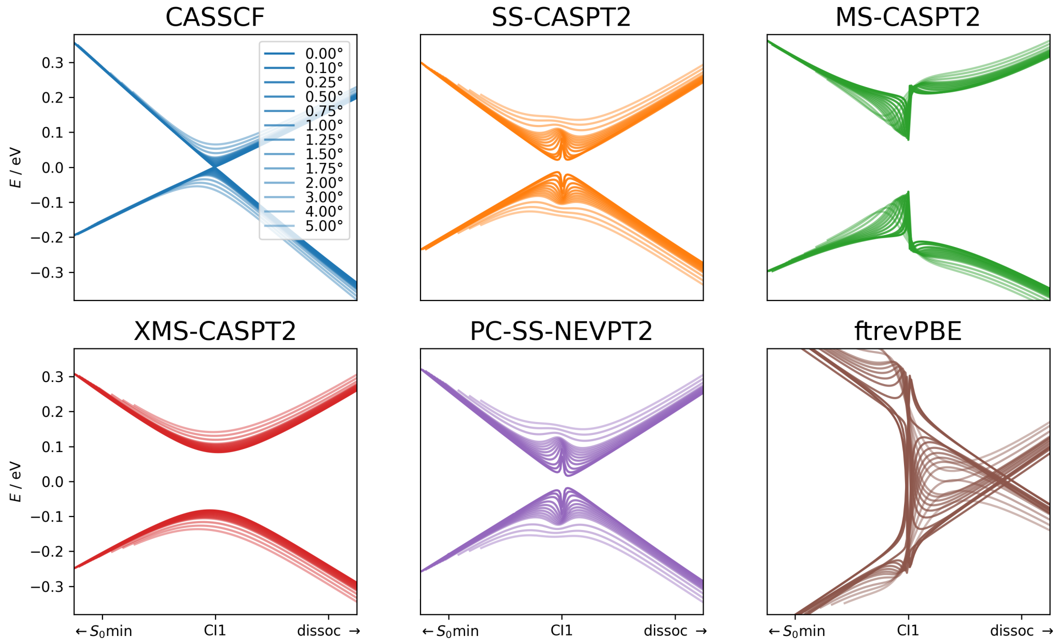

Figure S6. Series of scans of the potential energy surface passing around CI1 calculated with the SA-CASSCF, SS-CASPT2, MS-CASPT2, XMS-CASPT2, PC-SS-NEVPT2 and ftrevPBE methods. The more transparent the lines are, the further from CI1 the scan passes. The deviation from the exact CI in the dihedral angle of oxygen ranges from 0° to 5° at the reference SA-CASSCF level.

V. Fitting of populations and lifetimes

Here, we provide details about fitting the population curves and extracting lifetimes. The populations were fitted to the delayed exponential decay function

$$p_{\text{fit}}(t) = \begin{cases} 1 & \text{if } t \in [0, t_0] \\ \exp\left(-\frac{t-t_0}{t_1}\right) & \text{if } t > t_0 \end{cases} \quad (1)$$

as described in the main article. The fitted populations are plotted in **Figure S7** and the fit parameters are presented in **Table S2**. The delayed exponential function nicely fits the observed population traces mostly staying within the error bars.

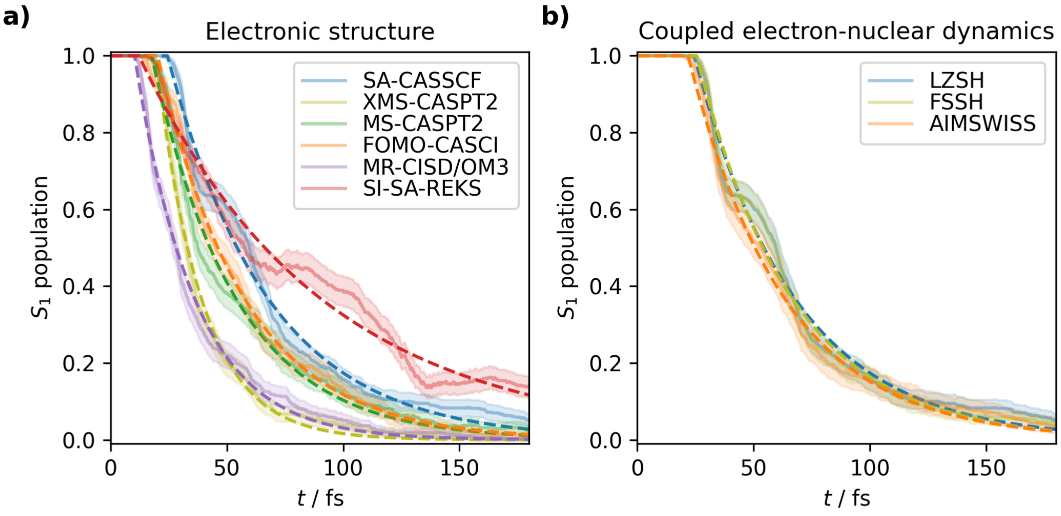

Figure S7. a) Populations of the S_1 state. b) Number of running simulations.

Table S2. Lifetimes of photodissociation for different methods of electronic structure and nuclear evolution.

	**method**	t_0 / fs	t_1 / fs	τ / fs
electronic structure	SA-CASSCF(8,7)	24.5 ± 4.6	43.2 ± 6.6	67.7 ± 4.6
	XMS-CASPT2(8,7)	19.5 ± 3.1	19.5 ± 4.3	39.1 ± 3.1
	MS-CASPT2(8,7)	17.7 ± 4.2	36.3 ± 6.0	53.9 ± 4.2
	FOMO-CASCI(8,7)	20.2 ± 4.3	37.3 ± 6.1	57.6 ± 4.3
	MR-CISD/OM3(8,7)	10.4 ± 3.5	25.8 ± 5.0	36.1 ± 3.5
	SI-SA-REKS(2,2)	12.2 ± 6.5	78.1 ± 10.1	90.3 ± 6.9
coupled el.-nuc. dynamics	LZSH	24.5 ± 4.6	43.2 ± 6.6	67.7 ± 4.6
	FSSH	25.6 ± 4.5	41.2 ± 6.4	66.7 ± 4.5
	AIMSWISS	21.9 ± 4.5	41.5 ± 6.4	63.4 ± 4.5

VI. Multidimensional scaling as a tool for the discovery of rare geometries

Here, we introduce MDS as an efficient tool for discovering rare geometries in big samples. As an example, we will take the FSSH dynamics with the SA-CASSCF(8,7) electronic structure. From the 600 trajectories, 575 hopping geometries were fetched and analyzed with MDS. Important points on the potential energy surface were included for better analysis. Only Cartesian coordinates of carbon atoms were processed in MDS. The oxygen was excluded for better visualization and hydrogens for decreasing noise. With these settings, the reduced coordinates were generated, see **Figure S8**.

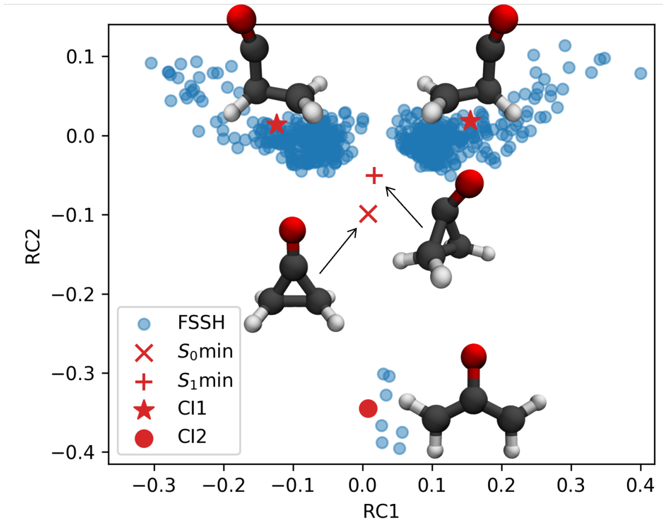

Figure S8. Hopping geometries from the FSSH dynamics with the SA-CASSCF(8,7) electronic structure plotted in reduced dimensions. RC1 corresponds to the opening of the ring next to the carbonyl group while RC2 corresponds to the opening of the ring opposite to the carbonyl.

RC1 corresponds to the opening of the ring next to the carbonyl group. The plane of symmetry of cyclopropanone is manifested in RC1. RC2 corresponds to the opening of the ring opposite to the carbonyl structure. This motion is symmetric itself, thus, no symmetry in RC2 is present. The hopping geometries are grouped into three clusters. The two big clusters in the upper part of the plot correspond to the transitions through CI1. These two clusters are separated by an empty region at RC1 equal to 0. This demonstrates that hopping never occurs with a closed structure but the ring must be always open to one side. However, the most interesting feature (from the perspective of this section) is the third small cluster in the lower part of the plot. It shows 7 geometries distinct from CI1. Taking these geometries as an initial guess, a new conical intersection coined CI2 was discovered. This conical intersection has not been reported before probably due to the fact that it appears very rarely – only in 7 samples from 575. While noticing CI2 by visual analysis of the hopping geometries would be time-consuming, MDS provides a straightforward detection without any visual inspection.

VII. The effect of active space on SI-SA-REKS

In order to better understand the failure of the SI-SA-REKS(2,2), we tested the effect of the smaller active space applied for the REKS results. We performed the SA-CASSCF(2,2) LZSH dynamics and compared it with SA-CASSCF(8,7) and SI-SA-REKS(2,2) results, see **Figure S9** and **Table S3**. The smaller active space prolongs the lifetime of SA-CASSCF dynamics up to 92.7 ± 6.7 fs which matches the 90.3 ± 6.9 fs of SI-SA-REKS. The quantum yield is also shifted to a lower value of 0.52 ± 0.04 closer to the SI-SA-REKS value (0.20 ± 0.03). Counting the polymerization pathway, we have observed no corresponding geometries for SA-CASSCF(2,2) which is in line with SI-SA-REKS(2,2). Comparing hopping geometries in **Figure S9b**, we observe the same pattern for SA-CASSCF(8,7) and SI-SA-REKS(2,2). Note that the RC1 and RC2 coordinates are not the same as for the plot in the article since they were produced by a separate run of MDS. Concluding, while the decrease in active space can explain the longer lifetimes, polymerization pathway counts and hopping distributions for SI-SA-REKS, it can only partially account for the very low quantum yield. Other aspects of the SI-SA-REKS method must play role.

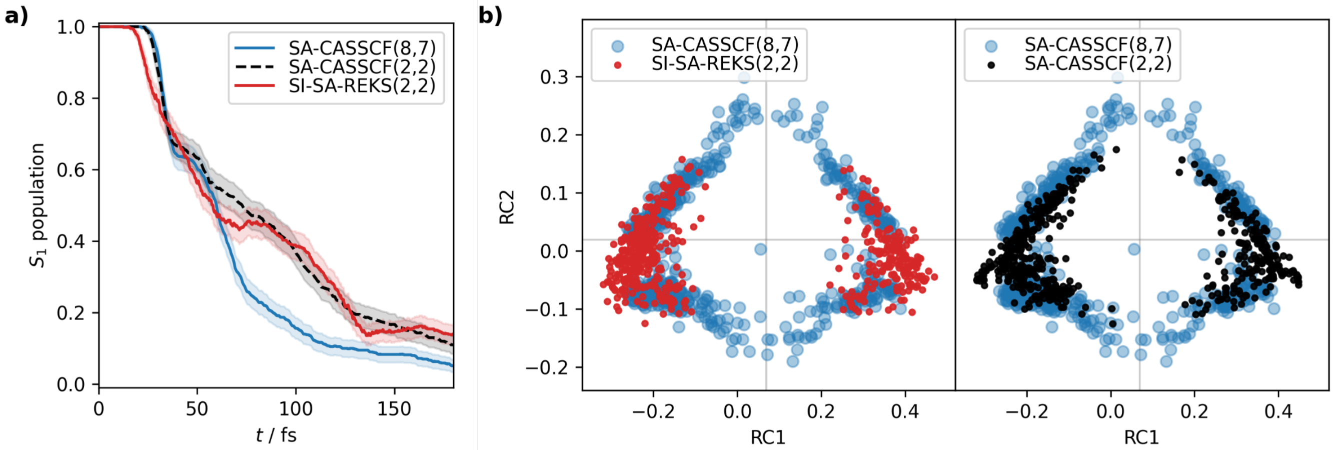

Figure S9. a) Populations of the S_1 state and b) hopping geometries processed by MDS for the SA-CASSCF(8,7), SA-CASSCF(2,2) and SI-SA-REKS(2,2) methods combined with LZSH.

Table S3. Lifetimes of the excited state (τ) together with fitting parameters (t_0, t_1), quantum yields of photodissociation (ϕ_{CO}) and counts of the polymerization pathway (N_{polymer}) for the SA-CASSCF(8,7), SA-CASSCF(2,2) and SI-SA-REKS(2,2) methods combined with LZSH.

**method**	t_0 / fs	t_1 / fs	τ / fs	ϕ_{CO}	N_{polymer}
SA-CASSCF(8,7)	24.5 ± 4.6	43.2 ± 6.6	67.7 ± 4.6	0.76 ± 0.04	4
SA-CASSCF(2,2)	18.0 ± 6.3	74.8 ± 9.9	92.7 ± 6.7	0.52 ± 0.04	0
SI-SA-REKS(2,2)	12.2 ± 6.5	78.1 ± 10.1	90.3 ± 6.9	0.20 ± 0.03	0

VIII. MS-CASPT2 vs XMS-CASPT2 in nonadiabatic dynamics

In the main article, we compare MS- and XMS-CASPT2 dynamics with LZSH and FSSH. Here, we provide more details like lifetimes with fitting parameters, quantum yields, and also counts of polymerization pathway, see **Table S4**.

Table S4. Lifetimes of the excited state (τ) together with fitting parameters (t_0, t_1), quantum yields of photodissociation (ϕ_{CO}) and counts of the polymerization pathway (N_{polymer}) MS- and XMS-CASPT2 combined with LZSH and FSSH.

**method**		t_0 / fs	t_1 / fs	τ / fs	ϕ_{CO}	N_{polymer}
LZSH	XMS-CASPT2	19.5 ± 3.1	19.5 ± 4.3	39.1 ± 3.1	0.78 ± 0.03	1
LZSH	MS-CASPT2	17.7 ± 4.2	36.3 ± 6.0	53.9 ± 4.2	0.76 ± 0.03	2
FSSH	XMS-CASPT2	19.0 ± 3.2	21.6 ± 4.6	40.7 ± 3.2	0.79 ± 0.03	1
FSSH	MS-CASPT2	18.3 ± 5.3	56.8 ± 7.8	75.1 ± 5.5	0.77 ± 0.04	2

References

(1) Suchan, J.; Hollas, D.; Curchod, B. F. E.; Slavíček, P. *Faraday Discussions* **2018**, *212*, 307–330.

(2) Rodriguez, H. J.; Chang, J.-C.; Thomas, T. F. *Journal of the American Chemical Society* **1976**, *98*, 2027–2034.

(3) Calvert, J. G.; Atkinson, R.; Kerr, J. A.; Madronich, S.; Moortgat, G. K.; Wallington, T. J.; Yarwood, G., *Mechanisms of Atmospheric Oxidation of the Alkanes*; Oxford University Press: 2008, pp 725–728.

(4) Angeli, C.; Cimiraglia, R.; Evangelisti, S.; Leininger, T.; Malrieu, J. P. *Journal of Chemical Physics* **2001**, *114*, 10252.

(5) Li Manni, G.; Carlson, R. K.; Luo, S.; Ma, D.; Olsen, J.; Truhlar, D. G.; Gagliardi, L. *Journal of Chemical Theory and Computation* **2014**, *10*, 3669–3680.

(6) Bao, J. J.; Zhou, C.; Varga, Z.; Kanchanakungwankul, S.; Gagliardi, L.; Truhlar, D. G. *Faraday Discussions* **2020**, *224*, 348–372.

(7) Werner, H.-J.; Knowles, P. J.; Knizia, G.; Manby, F. R.; Schütz, M. *WIREs Computational Molecular Science* **2012**, *2*, 242–253.

(8) Werner, H.-J. et al. *The Journal of Chemical Physics* **2020**, *152*, 144107.

(9) Aquilante, F. et al. *Journal of Chemical Physics* **2020**, *152*, 214117.

(10) Galván, I. F. et al. *Journal of Chemical Theory and Computation* **2019**, *15*, 5925–5964.
